# P04.85. Addressing nature deficit disorder: a quantitative survey study of multidimensional aspects of well-being among young adults at a wilderness camp

**DOI:** 10.1186/1472-6882-12-S1-P355

**Published:** 2012-06-12

**Authors:** S Warber, M Bialko, A Dehudy, K Irvine

**Affiliations:** 1 University of Michigan, Ann Arbor, USA; 2 De Montfort University, Leicester, United Kingdom

## Purpose

The health and well-being of America’s youth is increasingly in jeopardy due to a ‘screen-based’ culture that decreases exposure to the natural world. We hypothesized that interactions with nature would promote physical, psychological, emotional, and spiritual well-being. To investigate this claim, we surveyed young adults at a three-week science and wilderness camp in rural West Virginia.

## Methods

Online surveys were administered pre-camp to both campers and staff (n=46; 65% female, 35% staff, 18-31 years old, 51% from suburban/urban areas). Scales measuring nature experience were modified from previous research in camp settings. Validated, reliable instruments measured physical, psychological, emotional, and spiritual components of well-being, as well as nature connection. Completed post-camp surveys (n=36) were matched with pre-camp data and differences investigated using paired samples t-tests.

## Results

Statistically significant differences between pre- and post-camp experiences of the participants were identified on all nature-related measures: exposure (p<0.001), knowledge (p=0.018), skills (p<0.001), leadership willingness in a natural setting (p<0.001), sense of connection to nature (p<0.001), and sense of connection to place (p=0.001). All scores showed an increase post-camp. Several holistic well-being outcome measures also significantly improved: sense of wholeness (p=0.012), positive emotions (p<0.001), and experiences of transcendence (p=0.002) increased, while perceived stress (p=0.020) and negative emotions (p=0.003) decreased. However, physical activity level (p=0.084) and several psychological measures, including resilience (p=0.083), psychological well-being (p=0.943), self-esteem (p=0.950), self-awareness (p=0.200), and reflection (p=0.129), did not change.

## Conclusion

Findings demonstrate the profound change in relationship to nature that an emersion experience in wilderness can provide while also delineating elements of well-being (i.e., decreased stress, increased wholeness, more positive emotions, and more frequent spiritual experiences) that are affected by time spent in nature. Results can guide future research agendas and suggest that emersion experiences in nature could be prioritized in plans for improving and sustaining the health of America’s youth.

